# Identification of Pummelo Cultivars by Using a Panel of 25 Selected SNPs and 12 DNA Segments

**DOI:** 10.1371/journal.pone.0094506

**Published:** 2014-04-14

**Authors:** Bo Wu, Guang-yan Zhong, Jian-qiang Yue, Run-ting Yang, Chong Li, Yue-jia Li, Yun Zhong, Xuan Wang, Bo Jiang, Ji-wu Zeng, Li Zhang, Shu-tang Yan, Xue-jun Bei, Dong-guo Zhou

**Affiliations:** 1 College of Horticulture and Landscape Architecture, Southwest University, Chongqing, China; 2 Key Laboratory of South Subtropical Fruit Biology and Genetic Resource Utilization, Ministry of Agriculture, Guangzhou, China; 3 Institute of Tropical and Subtropical Cash Crops, Yunnan Academy of Agricultural Science, Dehong, Yunnan, China; 4 Institution of Fruit Tree Research, Guangdong Academy of Agricultural Sciences, Guangzhou, China; University of New England, Australia

## Abstract

Pummelo cultivars are usually difficult to identify morphologically, especially when fruits are unavailable. The problem was addressed in this study with the use of two methods: high resolution melting analysis of SNPs and sequencing of DNA segments. In the first method, a set of 25 SNPs with high polymorphic information content were selected from SNPs predicted by analyzing ESTs and sequenced DNA segments. High resolution melting analysis was then used to genotype 260 accessions including 55 from Myanmar, and 178 different genotypes were thus identified. A total of 99 cultivars were assigned to 86 different genotypes since the known somatic mutants were identical to their original genotypes at the analyzed SNP loci. The Myanmar samples were genotypically different from each other and from all other samples, indicating they were derived from sexual propagation. Statistical analysis showed that the set of SNPs was powerful enough for identifying at least 1000 pummelo genotypes, though the discrimination power varied in different pummelo groups and populations. In the second method, 12 genomic DNA segments of 24 representative pummelo accessions were sequenced. Analysis of the sequences revealed the existence of a high haplotype polymorphism in pummelo, and statistical analysis showed that the segments could be used as genetic barcodes that should be informative enough to allow reliable identification of 1200 pummelo cultivars. The high level of haplotype diversity and an apparent population structure shown by DNA segments and by SNP genotypes, respectively, were discussed in relation to the origin and domestication of the pummelo species.

## Introduction

Pummelo [*Citrus maxima* (J. Burman) Merrill] is an important cash crop that is widely cultivated and consumed in the world, and particularly in China and other Southeast Asian countries. Pummelo cultivars are easy to be mis-identified for the following reasons: first, there are hundreds of pummelo cultivars, and the origin and morphological traits of many local cultivars have been poorly documented and hence largely unknown to the outside world; second, pummelo cultivars have been traditionally distinguished by a few morphological traits, mostly fruit traits, and trees without fruit are often difficult to distinguish; third, unlike many other poly-embryonic species in *Citrus*, true pummelos are mono-embryonic and could have been both sexually and asexually propagated during domestication, adding more difficulty to the cultivar identification problem. A reasonable solution to the pummelo cultivar identification problem is to use DNA molecular data to help identify the cultivars.

Cultivated pummelos have been reported to be highly diverse in Southeast Asia, China and India by different researchers based on phenotypes of their fruits [1], [2], [3], [4]. Barkley et al. [5] analyzed 370 citrus accessions including 89 pummelo individuals on 24 SSRs and identified a heterozygosity of 0.4238 for the pummelo accessions. Traditionally, pummelo cultivars in China were classified into three groups, i.e., the Wendan group, the Shatianyou group and the hybrid group (interspecific). A study based on SSR and AFLP markers identified a high genetic diversity existing in the 110 analysed pummelo cultivars collected mainly from China, and showed that the Shatianyou group members were closely related to each other whereas members in Wendan group were more diverse [6]. In the study of Ollitrault et al. [7] using clementine (*Citrus clementina* Blanco) EST-SNPs, 10 pummelos were assigned to 8 different genotypes. These studies suggested that it should be possible to identify pummelo cultivars using molecular data.

SNPs are widely used for identity analysis in recent years [8], [9], [10]. SNPs were developed for variety identification in melon (*Cucumis melo* L.) [9], cereal [11] and capsicum (*Capsicum annuum* L.) [12]. Using SNPs in tree cultivar identification has also been reported. It was claimed that several thousands of grapevine (*Vitis vinifera* L.) cultivars could be distinguished with the use of a set of 48 SNPs [13]. Cultivar identification using SNPs was also used in olive (*Olea europaea* L.) [14] and *Eucalyptus* [15]. Another popular DNA marker in individual or cultivar identification is SSR [16], [17], [18]. Generally speaking, a SSR contains more polymorphic information content (PIC) than does a SNP, as SSRs are often multi-allelic while SNPs are mostly bi-allelic [19], [20]. A comparative study showed that a set of 23 selected SNPs was similar in discrimination power to a set of 13 SSRs for soybean (*Glycine max* (L.) Merr.) cultivar identification [8]. However, SNPs have several advantages over SSRs. First, the nomenclature of SNP is much simpler than that of SSR, which makes analysis and sharing of results much easier [21], [13]. Second, SNPs greatly outnumber SSRs in genomes, and the study of Tokarska et al. [20] showed that in case of difficult to obtain enough polymorphic SSRs due to low level of genetic diversity it was still possible to find enough number of SNPs for identity analysis. Third, SNPs are genetically more stable than SSRs, since SSR loci are prone to unequal recombination during meiosis or slippage during DNA replication, and new alleles or homoplasy can be generated [22], [23]. And SNP genotyping results have been reported to be highly consistent among different laboratories using different genotyping techniques [24].

High resolution melting analysis (HRMA) has been proven to be an efficient, moderately high throughput, and highly accurate genotyping method [25], [26]. HRMA allows tens to hundreds of samples to be analyzed in a single plate in 30 minutes or so depending on the type of machine used [27]. And these advantages make it quite suitable for quick identification of cultivars.

DNA segments represent another valuable method for genetic variability studies. Segments from mitochondrial, chloroplast or nuclear genomes have been utilized for species identification in DNA barcoding technology [28], [29], [30]. However, the segments used in barcoding are intentionally selected for their possessing enough inter-specific rather than intra-specific polymorphism and are therefore not so suitable for individual identification within a species. But in theory, DNA segments could still be applicable for identifying individuals or cultivars if enough independent polymorphic segments were used. In this respect, chloroplast and mitochondrial segments are less effective than nuclear DNA segments since they are inherited asexually [31]. In contrast, a polymorphic nuclear locus containing a number of alleles (haplotypes) could generate a larger number of genotypes in a diploid organism. Thus, combinations between different such loci would be numerous enough to allow any cultivar to be identified.

In this study, we tried to use SNPs and a limited number of DNA segments to achieve a quick and reliable identification of pummelos.

## Materials and Methods

### Plant materials

All the plant materials were acquired with permissions from their owners or preservers abiding by the laws in China and in Myanmar. Pummelo leaves collected from Myanmar were private possessions and the owners (Kareng ma kam, Changhai Leng, Kachin State, Myanmar) agreed with the use of the materials in research. The plant materials used in this study did not involve endangered or protected species.

Leaves of 205 citrus trees were collected from the National Citrus Germplasm Repository (Chongqing), and the Citrus Germplasm collection block (Guangdong) (Table S1). The samples included 99 pummelo cultivars, 26 unknown accessions, 24 hybrids between pummelo and other citrus species (referred to as CUL, UNKNOWN, MYANMAR and HYBRID respectively), a Honghe papeda (*Citrus hongheensis* Y. M. Ye, X. D. Liu, S. Ding, et M. Q. Liang) and a Honghe papeda hybrid. Two and three individuals were sampled for 30 and 12 cultivars respectively, which were marked in Table S1. Leaves from 55 pummelo trees were collected at the border between Myanmar and Yunnan province of China, of which 19 trees were located around N23°52.275′E97°41.285′and the rest 36 trees were located around N24°3.173′E97°35.349′. For convenience, all accessions and individuals will be collectively referred to as accessions. Genomic DNA was extracted from leaves using EasyPure Plant Genomic DNA Extraction Kit (TransGen Biotech, Beijing, China) using the protocol supplied with the kit.

### Identification and selection of pummelo SNPs

Two strategies were used to obtain pummelo SNPs. First, pummelo haplotypes were inferred from the EST sequences of sweet orange [*Citrus sinensis* (L.) Osbeck], sour orange (*Citrus aurantium* L.) and grapefruit (*Citrus paradisi* Macfayden) that are known to be derived from crosses between pummelo and other citrus species [32], [33], [34], [35], and the homologous haplotypes were compared pairwise to identify the pummelo intra-specific SNPs [36]. Second, direct sequencing of pummelo genomic segments was used to obtain more SNPs. Briefly, the genomic segments of *Zeaxanthin epoxidase* (*ZEP*), *Phytoene synthase* (*PSY*), *Phytoene desaturase* (*PDS*) and*β-carotene hrdroxylase* (*CHX*) genes that were 1604, 837, 764 and 2200 bp long respectively were cloned by PCR using the respective gene-specific primers from Guanximiyou and Lingnanshatianyou [36]. The PCR amplicons were then cloned using pEASY-T1 Simple Cloning Kit (TransGen Biotech, Beijing China). Two or more positive clones were sequenced for each gene segment from both cultivars using Sanger method. The synthesis of oligonucleotide primers and Sanger sequencing were carried out by Beijing Genomics Institute (BGI, Shenzhen, China). Sequences were aligned using Clustal X version 2.0 [37] and putative SNPs were identified by that the two SNP alleles should be represented by at least two different clones respectively.

A total of 60 putative SNPs were thus identified and used to select for a set of informative SNPs suitable for pummelo cultivar identification. First, each SNP was experimentally analyzed to exclude those that were unable to be genotyped by HRMA method. Second,allelic frequencies were estimated for each of the remaining SNPs by genotyping 24 randomly selected Chinese pummelo accessions (excluding somatic mutations), and the SNPs with minor allelic frequency >10% were retained as candidates. Third, all candidate SNPs were mapped onto the sweet orange reference genome [38] to estimate the physical distance and the linkage relationship between any two SNPs, and those without significant linkage to others were preferred. However, some physically linked SNPs were both retained for their high PIC values, and treated as super loci (described in Genotype analysis). Finally, a set of 25 SNPs were selected.

### SNP genotyping

High resolution melting analysis of small amplicons was used in SNP genotyping by following the protocol described by Gundry et al. [25]. LCGreen Plus+ Melting Dye (BioFire Diagnostics, Salt Lake, USA) was used, and HRMA was performed on 96-Well LightScanner System (BioFire Diagnostics, Salt Lake, USA). The fluorescence signal was recorded from 55°C to 90°C for all SNPs. The 3′ ends of the primers were designed to be as close as possible to the SNP loci so that the amplicons would be as short as possible [39], [40]. The primer sequences and lengths of the amplicons were listed in Table 1.

**Table 1:** HRMA Primer sequences and statistics on the 25 Set1 SNPs.

ID	SNP name	Accession No.	Forward primer	Reverse primer	Amplicon length (bp)	Ho	He	PIC	f
1	chr1_7539019A/G*	GO241840	ACATCACTCCACACACTAG	CGTTTTTATGATGGTCTAC	40	0.44	0.43	0.34	0.01
2	chr1_20043485A/G	EY724955	TGGTACTCAGGAATTTATATTA	AGCTTCTCCCAAGTCTT	50	0.27	0.29	0.25	0.07
3	chr1_25991020A/T	EY722700	AGAATTCCTGGTACCATC	AAGGATGATCCACGGT	36	0.31	0.32	0.27	0.01
4	chr2_31491T/C	GO241741	AAAAATCATCTCTGTGCAGC	CTCAATTCTGAAGCACATGAT	42	0.36	0.45	0.35	0.20
5	chr2_16534248A/G	GO242464	CCATCAAGTCATGAGTTTCTT	TAACTTTGTTCCCGGAAAGT	42	0.43	0.46	0.35	0.08
6	chr2_30594899T/C	EY661549	TGACTGCTGTGATTGTTCCT	ACAATTTCTGAACTATTGATATGTG	46	0.30	0.34	0.28	0.10
7	chr2_30595627T/C	EY656665	GCTCACCGAGAAAACTCTCC	GACGTTTCAAGTACATCACAATACA	61	0.25	0.30	0.25	0.16
8	chr3_10462341T/C	EY699140	CATGCTTAGGAAGTCTGT	CCTCTTTCGGCATTAC	35	0.19	0.37	0.30	0.49
9	chr3_25525170A/G	GO241356	GCCGGTACATTAACGTTTG	TTAGTTCATTCTACTGCATTTCAT	45	0.13	0.16	0.15	0.18
10	chr4_4533744A/G	GE213353	TATGGCGGACAAGTGATTG	TCAGCAGGAAAATCATTGAAC	41	0.40	0.49	0.37	0.18
11	chr4_14833122T/C	FC922642	TGATTGTAGGAGAAAGACGGA	ATTTCGTCTTTCAATAAAATCC	44	0.18	0.17	0.16	0.04
12	chr5_2097500T/G	FC922009	CCTTGTTTCTTCGCTTC	CCATTGTATGATCCGATC	37	0.43	0.49	0.37	0.14
13	chr5_12963514T/C	EY667938	ATACTCCATCACATTTGTGATCTC	AGAAAACAGTCAACTGTCAAGATG	51	0.38	0.36	0.29	0.05
14	chr5_13684450T/C	EY732058	TCGTTTTACAGTCCTATCTACAA	TGGGAGACCAGTCATTCTC	43	0.17	0.19	0.17	0.07
15	chr5_15275826A/G	HS085998	CGTGCAAGAAAACATCAATAG	TGTTTACTTTATGTGATTGTTGAAG	47	0.42	0.45	0.35	0.07
16	chr6_16520557A/G	HS086273	CTGAAGTGCACGTCCAAAG	AAGCCATGGTCACTTTCCTT	42	0.41	0.49	0.37	0.16
17	chr7_7194644A/G	JZ120109	GCTGATTCTTATGGATCAACT	GCATTACCAGTCAATGTATTG	43	0.17	0.19	0.17	0.07
18	chr7_7195033T/G	GO241763	GACGCTAAGGCACGCCG	ATCAACTGCAAACCAGCAAAA	39	0.17	0.22	0.20	0.21
19	chr7_22205388T/C	HS088559	CCAGAACATTTCATTGATGC	GGAAGGGGTCTAAGTCAGG	40	0.24	0.27	0.23	0.10
20	chr7_31599886T/G	GO242250	GCTCTTGGTGAAGCTAATG	ATTTCCACTCTGAATAGCATCT	42	0.10	0.11	0.10	0.14
21	chr9_2379630T/G	HS088445	AAATGTTGGGTCGGTCG	CTTTGCTTACTACAAGGAAGGT	40	0.30	0.50	0.37	0.39
22	chr9_13716551T/C	EY688408	CAGCAATCCCGGCAGCT	CTCCTCTACATTATAAGCTTGGTCA	43	0.10	0.12	0.11	0.13
23	chr9_15596085A/G	JK694395	GGAACCAGAGAGTGTGAAGAT	GGCTCGAATTTGGACTGA	41	0.42	0.39	0.32	0.05
24	chrUn_5023005A/G	HS086380	AAATGTGAGGTTCCATAAGGA	ACTCTGGGTCATTCTTCTCTTC	44	0.47	0.48	0.37	0.03
25	chrUn_19904498A/G	GO241683	CTCTAGACTGCCAGCTTCATAG	TGGACCGGGTTTTATCAGAT	44	0.43	0.38	0.31	0.11
Mean	-	-	-	-	-	0.30	0.34	0.27	0.11

*The SNP name chr1_7539019A/G: an A/G SNP at the 7539019th bp on the pseudo-chromosome 1 of the sweet orange reference genome, and et cetera. Ho, observed heterozygosity; He, expected heterozygosity; PIC, polymorphic information content; f, fixation coefficient.

Genotyping by direct sequencing of PCR amplicons was used to a) verify the HRMA results, b) identify possible primer-template mismatches encountered in HRMA analysis, and c) genotype the gene segments. PCR primers were designed so that the amplicons would be around 500 bp long with the known SNP in the middle to ensure that high quality sequencing data surrounding the SNP would be obtained by Sanger sequencing method that was used in this study. For identifying primer-template mismatches, primers were compared with their complementary sequences of the templates.

For the 12 sequenced gene segments, SNPs were detected and all genotypes were read out from sequencing data by using Variant Reporter Software v1.1 (Applied Biosystems, Foster City, CA), and the genotypes were visually examined by reading sequence chromatograms.

### Genotype analysis

The total number of genotypes and the common genotypes shared by two or more cultivars were analyzed using Dropout [41]. The distribution of the minimum pairwise differences between a genotype and other genotypes was shown by Dropout. All pummelo accessions/individuals were grouped, either by geological origins where the samples were collected (MYANMAR group) or by traditional classification (SHATIANYOU group and WENDAN group) (Table S1). The fixation coefficient was calculated for each group using GenAlEx [42]. The relationship showing that the increase in genotype numbers with the adding of SNPs was displayed by GenAlEx. To show population structure, a Bayesian clustering method was applied to the whole dataset to assign genotypes to inferred clusters using STRUCTURE version 2.3.4 [43]. Ten independent runs of K = 1 to 8 each were performed at 1,000,000 Markov Chain Monte Carlo (MCMC) repetitions with a 100,000 burn-in period using no prior information and assuming correlated allele frequencies and admixture. The *ln* likelihood of the posterior probability K [P (K|X)] was used to choose the most likely value for K.

The statistical power in identity analysis was calculated for the set of 25 SNPs used in the total sample and in different groups or populations respectively. The level (r^2^) of linkage disequilibrium (LD) between SNPs was analyzed using PowerMarker V3.25 [44]. SNPs in significant LD (r^2^>0.1, p<0.05) were combined as a super locus, and haplotypes and haplotype frequencies were inferred using PowerMarker V3.25. The probability of identity between two random individuals (PI), between two random sampled siblings (PIsibs), and between a random selected pair of a parent and an offspring (PIpar-off) were calculated using GenAlEx [42], [45], [46], [47]. The PI, PIsibs and PIpar-off for each of the 178 genotypes identified from the total sample were also calculated using Dropout [41].

For the 12 gene segments, segregating sites (S), nucleotide diversity (π) and haplotype diversity (Hd) were obtained by using DnaSP v. 5.10.01 [48]. Haplotypes were reconstructed from genotype data using DnaSP v. 5.10.01. PI and PIsib for the 12 gene segments were calculated using GenAlEx [42].

The number of cultivars (n) which could be reliably discriminated by the selected SNPs or DNA segments was calculated using the following formula: 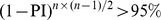
where PI is the total PI of the selected SNPs or DNA segments. This formula assures that in a sample of n cultivars, the chance for the non-existence of any false identical genotypes (two different genotypes were identified to be the same using the marker set) is larger than 95%.

### Construction of Neighbour-joining (NJ) tree

The haplotypes inferred from the 12 gene segments were used to construct Neighbour-joining tree together with the corresponding sequences of the haploid sweet orange genome [38], the haploid clementine genome [49] and Nanju (*Citrus reticulata* Blanco var. Nanju) whole genome resequencing data (our unpublished data) using PAUP 4.0 [50]. The evolutionary distances were computed using the Tajima-Nei method [51] and trees were displayed in FigTree V1.40 [52].

## Results

### Identification of pummelo SNPs and genotyping of pummelo accessions by HRMA

A total of 60 putative pummelo SNPs were obtained using the two strategies described in materials and methods, and out of them, a set of 25 SNPs (hereafter referred as Set1 SNPs) were selected and used to identify pummelo cultivars (see in materials and methods). Mapping on the sweet orange reference genome showed that these SNPs are interspersed on 8 of the 9 citrus (2n = 18) chromosomes (Table 1) except two that were located on scaffolds that have not been assigned to any chromosome.

**Figure 1:**
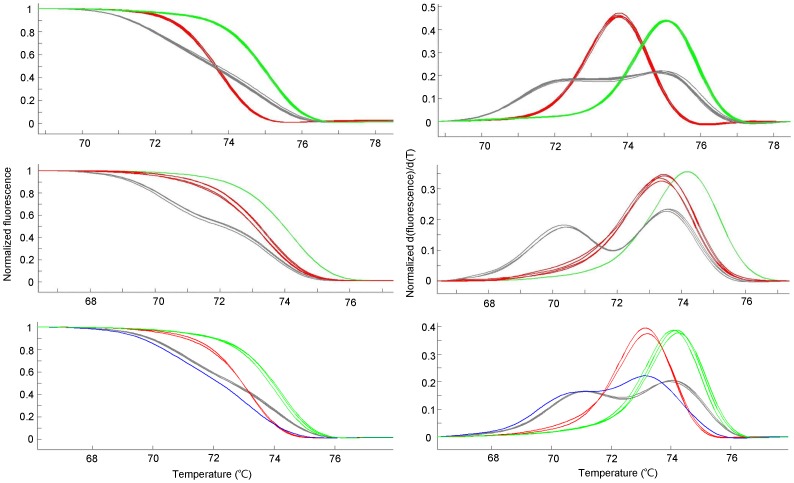
Representative high resolution melting curves for pummelo SNPs. The left and the right panels were melting curves and derivative melting curves respectively. Red and green curves were homozygous amplicons with low and high melting temperatures, respectively. Gray and blue curves represent heterozygotes. Top panels: melting curves of an A/G SNP (chr1_20043485A/G). Middle panels: melting curves of an A/T SNP (chr1_25991020A/T). Bottom panels: melting curves of an A/G SNP (chr4_18094735A/G); note the two different heterozygotes with different melting curves, the normal heterozygote (referred as He1) as shown in gray and the abnormal heterozygote (referred to as He2 that probably contained a primer-template mismatch) in blue.

The genotypes of the pummelo accessions were revealed by using high resolution melting analysis of amplicons. On all the 25 Set1 SNPs, heterozygotes were clearly distinguishable from homozygotes by shapes of their respective melting curves and derivative melting curves. The Tm difference between the two different homozygote amplicons of each SNP was between 0.6 and 2.0°C, and accordingly, the amplicons' melting and first derivative curves were shifted away from each other along the horizontal axis (Figure 1).

HRMA of small amplicons was supposed to be less powerful in distinguishing different homozygotes of A/T or G/C SNPs since there is no difference in hydrogen-bonds between the two homozygotes. However, the SNP chr125991020_A/T which was an A/T SNP showed ∼1°C of difference in Tm values between the two different homozygotes, making them clearly distinguishable (Figure 1). Currently, we have no explanation for this.

To our surprise, two or more different heterozygous melting curves were observed on 3 SNPs during our initial SNP screening work as shown by SNP chr4_18094735A/G in Figure 1. It was suspected that there existed extra mutation(s) in primer-template complementary regions, which made the PCR amplification favor the template that paired perfectly with the primers because any mismatch between template and primer would reduce the chance of the primer annealing to the template. Apparently, the majority of the amplicons should be from the template pairing perfectly with the primers rather than from that pairing imperfectly with the primers, and the shape of the resulting melting curve should thus be distorted so as to approach to the typical curve of the amplicons from the homozygous template without primer-template mismatches (Figure S1). To verify, the flanking sequences of the 3 SNPs were cloned and sequenced, and new SNPs were indeed found on their primer-template pairing regions (Figure S1). The three SNPs were therefore excluded from further use.

To find how reliable our HRMA genotyping results were, 36 cultivars were repeatedly analysed (two to three times) at all Set1 SNP loci, and a total of 6 mismatches were identified. Detailed analysis indicated that these mismatches were resulted from experimental errors, i.e., sample loading errors, which were identifiable and correctable by either repeating HRMA analysis or inspecting manually the melting curves. In addition, genotyping by sequencing was also done for 1∼25 accessions at Set1 SNP loci to verify the HRMA data. In the end, it was found the results from both sequencing and HRMA were fairly consistent.

### Genotypes of the 260 pummelo accessions

**Figure 2:**
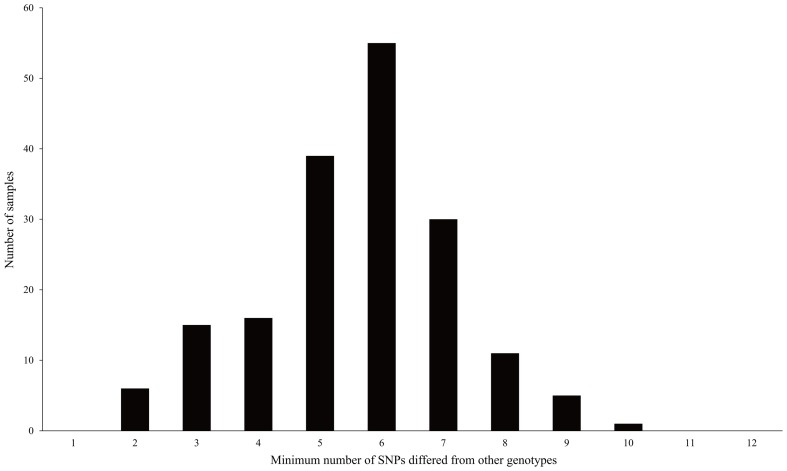
Distribution of the minimum number of SNPs differed between a genotype and other genotypes.

**Figure 3:**
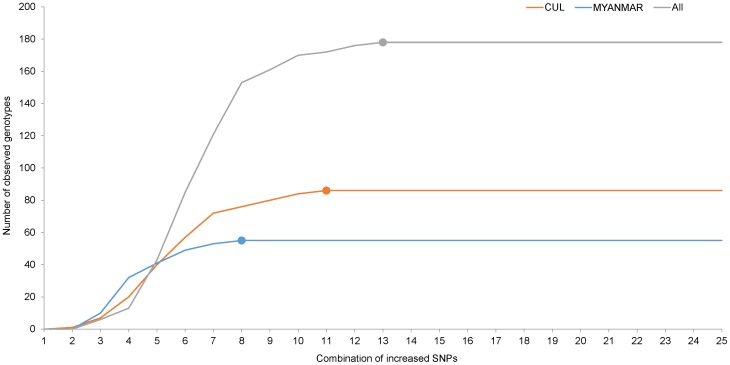
The relationship between observed genotypes and numbers of used SNPs. ALL, all the 178 genotypes; MYANMAR, the 55 Myanmar genotypes; CUL, the 86 cultivar genotypes. The 25 SNPs were ranked, from large to small, by their PIC values in ALL, MYANMAR and CUL, respectively, and then added one by one into analysis.

Genotyping of the 260 accessions were conducted on all Set1 SNPs (Table S1). As a result, a total of 178 different genotypes were identified (Table S1). It was shown that the minimum difference between a genotype and any of the other genotypes reached an average of 6 SNPs and the smallest was 2 SNPs (Figure 2). Most of the samples with the same genotypes were found to be individuals of the same cultivars or cultivars derived from somatic mutations. Synonyms were identified, including Gaopoyou/Bianboyou, Guanxiangyou/Pengxiyou, and Jinshayou/Baiyushuang. CUL were assigned to 86 different genotypes. For 6 cultivars, the samples collected from Chongqing did not match those collected from Guangzhou. Eleven of the 26 UNKNOWN accessions matched known cultivars, and the remaining 15 accessions were assigned to 15 new genotypes. The 55 MYANMAR individuals were assigned to 55 unique genotypes. The 24 HYBRID accessions were assigned to 20 different genotypes since red Hassaku and Hassaku were identified to be the same genotype, and so were Beni Amanatsu and Kawano Natsudaidai, Red Marsh, Star Ruby and Flame grapefruits. Honghe papeda and its hybrid were assigned to 2 unique genotypes. It was shown in Figure 3 that the number of genotypes identified increased with increased use of SNPs and 13 SNPs were already enough to distinguish all 178 genotypes. Only 11 and 8 SNPs were needed to identify all the CUL genotypes and the MYANMAR individuals, respectively.

From this study, a DNA fingerprint database was established for the analyzed pummelos (Table S1), and can be used as references when a cultivar needs to be typed.

### Population structure analysis and Statistical Power of SNPs in cultivar identification

The 25 SNPs have an average PIC value of 0.271 in the total sample (Table 1). The same set of SNPs could have different power in identity analysis in different populations, since populations are often different in allelic frequencies. According to tradition, the 101 CUL+UNKNOWN genotypes (86 cultivar genotypes +15 unknown genotypes) were classified into SHATIANYOU group (7 genotypes), WENDAN group (25 genotypes) and UNASSIGNED group (69 genotypes). To detect if there truly was population structure in pummelos the genotype data were subjected to population genetics analysis. Test against Hardy–Weinberg equilibrium showed that the null hypothesis was rejected (p<0.05) on 9 and 3 of the 25 Set1 SNPs in CUL+UNKNOWN group and in MYANMAR group, respectively, suggesting the accessions in the two arbitrary assigned groups could be in fact from different populations. In addition, the fixation coefficients of the SHATIANYOU group and the WENDAN group were 0.02 and 0.10, respectively, suggesting also that there could exist unresolved population structure in WENDAN group.

By using Bayesian clustering program STRUCTURE version 2.3.4 with K = 1∼8, the average ln likelihood values peaked at K = 4 for either the CUL+UNKNOWN or the CUL+UNKNOWN+MYANMAR, indicating most likely there were 4 populations in our analyzed samples. The 4 supposed populations of the CUL+UNKNOWN+MYANMAR had a population differentiation value of 0.16 (Fst), and were designated as P1, P2, P3 and P4. And 29, 46, 38 and 43 of the 156 CUL+UNKNOWN+MYANMAR genotypes were clustered into P1, P2, P3 and P4, respectively (Figure S2).

**Figure 4:**
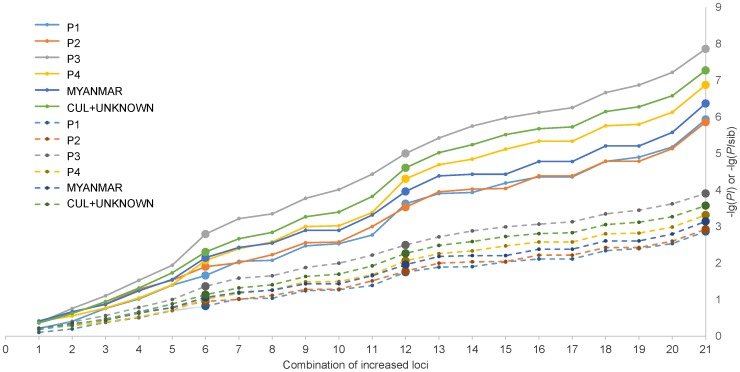
Increases in –lg(PI) and –lg(PIsib) with the use of increasing number of SNPs in different populations or groups. SNPs were added one by one in the order from 1 to 25 (listed in Table 1) in the construction of the plot. (chr2_30594899T/C, chr2_30595627T/C), (chr5_12963514T/C, chr5_13684450T/C, chr5_15275826A/G), and (chrUn_5023005A/G, chrUn_19904498A/G) were treated as three super loci (the 6^th^, 12^th^ and 21^st^ dots (large)). The solid and dash lines connect PIs and PIsibs, respectively.

Significant LD (r^2^>0.1, p<0.05) was discovered in three groups of SNPs. The first group contained two SNPs, chr2_30594899T/C and chr2_30595627T/C, which were closely spaced on chromosome 2. The second group contained three SNPs, chr5_12963514T/C, chr5_13684450T/C, and chr5_15275826A/G, that located on chromosome 5. The third group, chrUn_5023005A/G and chrUn_19904498A/G were on different scaffolds having not yet been assigned to any of the sweet orange chromosomes. The 3 groups of SNPs were therefore regarded as three super loci, and hence the 25 Set1 SNPs were actually treated as 21 loci in our statistical analysis (Figure 4).

When using the Set1 SNPs in CUL+KNOWN and MYANMAR, we obtained a PI value of 5.28E-08 and 4.25E-07, respectively, indicating the discriminating power of Set1 SNPs was different between the two groups (Figure 4). Comparison between the 4 populations showed that the strongest discrimination power of the SNP set was in P3 while the weakest was in P2. The PI values in P3 and P2 were different by two magnitudes as shown in Figure 4, which were 1.36E-08 and 1.38E-06, respectively. However, theoretical calculation showed that the Set1 SNPs was still powerful enough to be used for identity analysis in a population of 1000 individuals (N) with similar to P2's allelic frequencies. The power of this set of SNPs in discriminating siblings was given by PIsib (Figure 4), which varied from the strongest (1.24E-04) in P3 to the weakest (1.35E-03) in P2, suggesting the set of SNPs could discriminate 40 to 150 siblings. The PI, PIsib and PIpar-off for each of the 101 CUL+UNKNOWN genotypes were given in Table S2. For parentage exclusion with 200 candidate pummelo parents [53], the set of SNPs was shown to be not strong enough, and two to three times more SNP loci should be needed to be statistically powerful enough.

### Use of DNA sequences for pummelo cultivar identification

Twelve gene segments were sequenced for 24 accessions (Table 2). The lengths of the sequenced gene segments were between 410–630 bp and totaled at 6107 bp. On these sequenced segments 127 reliable SNPs were identified. Haplotype profiles were obtained for all the 24 accessions based on these SNPs (Table 2). As shown in Table 2, the 24 accessions were assigned to 23 distinct genotypes, which was consistent with the result by genotyping the Set1 SNPs. Notably, Hejiangyou and Lingnanshatianyou were identified as the same genotype by both methods. Since Boluoxiangyou, Tangyouzi, Jiaodaoyou and Iwaikan, were shown by phylogenetic tree to contain various numbers of mandarin haplotypes (Figure S3) they were treated as hybrids and excluded from analysis for pummelo intra-specific SNPs. Within the remaining sequences we identified 54 pummelo intra-specific SNPs (hereafter referred as Set2 SNPs) (Table 3).

**Table 2:** Genotypes of the 24 sequenced accessions.

Group	Gene ID	Cs1g16760	Cs1g23450	Cs2g19680	Cs2g31250	Cs4g07130	Cs4g15590	Cs5g15460	Cs7g10980	Cs7g31800	Cs9g04300	Cs9g14320	Cs9g16170
	Genomic locations of sequenced segments	chr1:20042999–20043567	chr1:25990710–25991189	chr2:16533735:16534365	chr2:30595507–30595981	chr4:4533481–4533998	chr4:14833018–14833473	chr5:13684338–13684914	chr7:7194703–7195183	chr7:31599722–31600139	chr9:2379414–2379892	chr9:13716462–13717019	chr9:15595778–15596191
1	Dayongjuhuaxin	(1,2)*	(2,5)	(1,1)	(5,5)	(3,5)	(2,2)	(4,7)	(5,10)	(1,1)	(4,5)	(1,2)	(1,2)
1	Fujianwendan	(1,2)	(5,5)	(2,4)	(2,4)	(3,3)	(1,2)	(4,6)	(7,9)	(1,1)	(1,2)	(1,6)	(3,4)
1	Guanximiyou	(2,2)	(5,5)	(1,1)	(4,5)	(1,2)	(1,2)	(4,6)	(6,6)	(1,3)	(1,2)	(1,1)	(4,4)
1	Liangpingyou_cq	(1,2)	(2,2)	(1,2)	(5,5)	(3,3)	(1,2)	(4,4)	(6,6)	(1,3)	(1,1)	(1,1)	(4,4)
2	Dongguaquan	(2,3)	(2,5)	(2,2)	(5,5)	(3,4)	(2,2)	(4,7)	(6,8)	(1,3)	(3,4)	(6,7)	(3,6)
2	Hejiangyou	(2,3)	(5,5)	(2,2)	(5,5)	(3,4)	(1,2)	(4,4)	(6,8)	(1,1)	(4,7)	(6,7)	(3,6)
2	Hongxinshatianyou	(2,2)	(2,5)	(2,4)	(5,5)	(3,4)	(1,1)	(7,7)	(8,10)	(1,1)	(4,7)	(5,5)	(3,4)
2	Lingnanshatianyou	(2,3)	(5,5)	(2,2)	(5,5)	(3,4)	(1,2)	(4,4)	(6,8)	(1,1)	(4,7)	(6,7)	(3,6)
2	Meiweishatianyou	(2,2)	(5,5)	(2,2)	(5,5)	(4,4)	(1,2)	(4,7)	(6,8)	(1,1)	(7,7)	(5,7)	(2,3)
2	Zhaipoyou	(2,3)	(5,5)	(1,2)	(5,5)	(2,3)	(2,2)	(4,7)	(6,8)	(1,1)	(1,7)	(1,6)	-
3	Zuoshiyou	(1,2)	(2,5)	(1,1)	(4,5)	(3,4)	(1,1)	(4,4)	(2,10)	(1,1)	(4,4)	(1,6)	(4,6)
3	Unknown	(1,2)	(2,5)	(1,2)	(4,5)	(1,2)	(1,1)	(4,5)	(6,10)	(1,1)	(1,2)	(1,9)	(2,4)
3	Dianjianghongxinyou	(1,2)	(2,2)	(1,2)	(5,5)	(1,4)	(1,1)	(7,7)	(4,10)	-	(4,4)	(1,6)	(6,6)
3	Duanshiyou	(2,3)	(5,5)	(1,2)	(5,5)	(1,4)	(1,1)	(4,4)	(6,6)	(1,1)	(4,4)	(4,9)	(3,4)
3	HB	(3,4)	(5,5)	(2,2)	(5,5)	(1,3)	(1,2)	(4,4)	(2,4)	(1,1)	(4,4)	-	-
3	Humiyou	(2,2)	(2,4)	(2,2)	(3,5)	(3,3)	(1,2)	(4,7)	(3,10)	(1,1)	(1,4)	(1,3)	(4,4)
3	Qiyou	(3,3)	(5,5)	(1,2)	(5,5)	(4,4)	(1,1)	(4,7)	(6,8)	(1,1)	(4,4)	(4,4)	(2,3)
3	Shuhuayou	(2,2)	(2,5)	(1,2)	(5,5)	(3,4)	(1,2)	(4,7)	(6,10)	(1,1)	(7,7)	(5,7)	(3,4)
3	Taiguomiyou	(2,3)	(2,3)	(1,1)	(4,5)	(3,4)	(2,2)	(4,4)	(2,10)	(1,1)	(4,4)	(1,7)	(2,3)
3	Xianluodisuanyou	(1,2)	(5,5)	(1,1)	(5,5)	(3,4)	(1,1)	(4,4)	(10,10)	(1,3)	(4,7)	(1,1)	(4,4)
4	Boluoxiangyou	(1,1)	(1,2)	(2,3)	(1,5)	(1,4)	(1,3)	(1,4)	(1,10)	(1,3)	(1,1)	(1,3)	(4,5)
4	Jiaodaoyou	(1,2)	(5,5)	(2,3)	(6,7)	(1,6)	(2,4)	(4,5)	(10,10)	(1,1)	(6,8)	(1,6)	-
4	Tangyouzi	(2,4)	(1,5)	(2,3)	(5,9)	(1,6)	(1,3)	(2,4)	(1,5)	(2,4)	(6,6)	(3,8)	-
4	Iwaikan	(1,3)	(5,5)	(2,3)	(5,8)	(3,6)	(1,3)	(2,3)	(1,6)	(2,4)	(4,4)	(3,3)	(5,5)

Numbers in parenthesis represent the two haplotypes of a gene segment in a cultivar. Different numbers represent different haplotypes; -, data unavailable. Group 1, Wendan; group 2, Shatianyou; group 3, hybrids; group 4, unassigned accessions. Gene ID: Cs1g16760, 40S ribosomal protein SA; Cs1g23450, Oxygen-evolving enhancer protein 1; Cs2g19680, Chlorophyll a–b binding protein of LHCII type I; Cs2g31250, Serine–glyoxylate aminotransferase; Cs4g07130, Protein TIFY 10A; Cs4g15590, Putative uncharacterized protein Sb09g005910; Cs5g15460, Protein RecA; Cs7g10980, Agamous-like MADS-box protein AGL9 homolog; Cs7g31800, Ribulose bisphosphate carboxylase/oxygenase; Cs9g04300, Translationally controlled tumor protein like protein; Cs9g14320, DC 1.2-like protein; Cs9g16170, Putative uncharacterized protein At5g6220.

**Table 3:** Nucleotide diversity and haplotype diversity in the 20 sequenced true-to-type pummelos.

Gene Accession Number	Cs1g16760	Cs1g23450	Cs2g19680	Cs2g31250	Cs4g07130	Cs4g15590	Cs5g15460	Cs7g10980	Cs7g31800	Cs9g04300	Cs9g14320	Cs9g16170	Mean
Available sequences	40	40	40	40	40	40	40	40	38	40	38	36	39.33
Segment length (bp)	569	499	631	475	518	456	577	473	454	479	558	410	508.25
Number of SNPs	2	3	2	5	5	3	3	6	1	6	10	8	4.5
Number of Haplotypes	4	4	3	4	5	2	4	9	2	6	8	5	4.7
Haplotype diversity	0.57	0.50	0.56	0.31	0.69	0.50	0.53	0.77	0.19	0.69	0.80	0.75	0.57
Nucleotide diversity	0.0012	0.0012	0.0010	0.0028	0.0032	0.0033	0.0010	0.0049	0.0004	0.0039	0.0061	0.0060	0.0029

For the 20 true to type pummelo cultivars remained, the nucleotide diversity (*π*) of the 12 gene segments varied from 0.0004 to 0.0061 and averaged at 0.0029 (Table 3). The number of identified haplotypes on each gene segment varied from 2 to 9 and averaged at 4.7 haplotypes/segment, and the haplotype diversity (equivalent to PIC) varied from 0.31 to 0.80 and averaged at 0.57 (Table 3), which was more than twice of the average PIC value of the Set1 SNPs. Significant LD was discovered between two linked gene segments (Cs9g14320 and Cs9g16170) mapped on pseudo-chromosome 9 of the reference sweet orange genome, and the two segments were combined as a super locus in PI calculation. PI and PIsib of the 12 segments were 4.9E-08 and 8.7E-04, respectively, suggesting that the combined use of the 12 gene segments is very powerful for pummelo cultivar identification but has limited power in distinguishing siblings. It was found that the haplotype diversity for each segment was significantly (p<0.01) correlated with (by Pearson correlation analysis, r^2^ = 0.53) the number of SNPs found on the segment (varied from 1 to 10 SNPs).

## Discussion

Cultivar identification is a prerequisite for a more efficient breeding activity and a more successful cultivation for a crop. The problem is that it is often difficult to identify cultivars solely by morphological traits [54]. This is because commercial cultivars are more or less similar to each other in many agronomic traits that have been convergently selected by humans. Though many methods have been explored, it seems that only DNA markers offer a satisfactory solution to this problem since suitable number of DNA markers, if used collectively, have been demonstrated to be good enough to identify non-clonal individuals or plant varieties [8], [9], [16], [17], [18]. For example, a bi-allelic SNP locus can have three possible genotypes: 2 homozygous and 1 heterozygous, and N such SNP loci can have 3^N^ different combinations, a number increases exponentially with increase in N. If N is large enough, the possible combinations will also be large enough to accommodate all known sexually originated cultivars. In other word, the combinations of a suitable number of DNA markers can be used as genetic barcodes to be assigned to a definite number of cultivars. The advantages of such a cultivar identification system are two-folds: 1) the barcodes can be easily recorded and shared; 2) the identification results are rather precise with statistical support. With such a system, to identify a cultivar needs only to analyze its genotypes at the loci used for genetic barcodes and compare the results to those in the database. In this study we investigated the possibility of using combinations of either SNPs or DNA segment sequences in the identification of pummelo cultivars that are easily misidentified by morphological traits, and to our satisfaction, both methods were shown to be powerful enough to identify our study samples. Therefore, the SNP genotypes listed in Table S1 can be used as reference genetic barcodes for pummelos.

High PIC and low level of LD between markers have been the two important factors to be considered in selecting markers for high efficient identification of cultivars [8], [13]. PIC was largely dependent on allelic frequency. To increase PIC some researchers used only SNPs with higher than 0.1 allelic frequency [55], and some others set the PIC value higher than 0.2 when choosing markers [8]. In our case, with the use of SNPs with an allelic frequency of higher than 0.1, a relatively satisfactory PIC value was obtained. LD not only reduces the overall discrimination power of the markers used [8], [56] but also influences the evaluation for discrimination power of the markers. Nevertheless markers physically linked could still be used in parentage and identity analysis since the linkage phase could be assessed and the tightly linked SNPs could be used as a ‘super locus’ [57], [58]. Such super locus could still have a very high PIC as shown in the present study and other studies [58]. However, markers in significant LD are not necessarily physically linked, since LD could also be caused by other factors such as genetic drift, bottle neck effect, selection within populations, and population admixture et al. [59]. In our study, two of the Set1 SNPs, chrUn_5023005A/G and chrUn_19904498A/G, were in LD but most probably were not physically linked.

The transferability of the SNPs discovered from certain samples to other samples may be limited, which is known as ascertainment bias [60], [61]. Ascertainment bias could influence the use of SNPs in cultivar identification. This is because SNPs highly polymorphic in one population are not necessarily similarly polymorphic in other populations. Anyway, the problem could be solved at the stage of SNP discovery. An intuitive way is to select ancient SNPs that are homogeneously polymorphic among the study populations as demonstrated in 6 *Eucalyptus* species by Correia et al. [15]. In a case of human individual identification, small *F*st values (<0.06 in the study of Pakstis et al.) were required for SNPs in order to avoid ascertainment bias [10]. In this study, ascertainment bias was shown to influence the PI value of Set1 SNPs by at most two magnitudes, even though, the set of the SNPs should be still usable in a predicted population of N = 1000 with the lowest PI value. Therefore, the barcode system will have enough room for accommodating future cultivars.

Taking together, the Set1 SNPs should be very usable for pummelo cultivar identification. And this conclusion was verified by the genotyping results of the 260 accessions. First, only 13 SNPs were needed to discriminate all the discovered genotypes (Figure 3), and every two genotypes were different by at least 2 SNPs when all the 25 SNPs were used (Figure 2), showing that this set of SNPs were more than enough for the discrimination of all study genotypes. Second, accessions known to be different were discriminated, and all the 55 Myanmar individuals grown from seedlings were assigned to 55 different genotypes. Third, the obtained PI values also suggested that the 25 Set1 SNPs were powerful enough for identification of pummelo cultivars.

The accuracy of the genotyping method influences the efficacy of cultivar identification [62]. In this respect, HRMA has been shown to be highly efficient and accurate [25], [36], [40], [63], [64], [65]. In the study of Gundry et al. [25], the accuracy of HRMA reached 99.7%. However, mismatches between template and primer reduced greatly the accuracy as shown in this study (Figure 1 and Figure S1), and therefore, should be avoided. It was noted that even a 5′ end template-primer mismatch distorted the melting curves severely enough to interfere with judgment about the genotype (Figure 1), and more severe distortion with 3′ end mismatches should be expected.

In due course of verifying the Set1 SNP genotyping results by sequencing, we noted that a high haplotype diversity existed in the sequences. This prompted us to investigate if it was possible to use directly the gene sequences to identify cultivars, which is similar to the traditional DNA barcoding technology used in identifying species. We set out to sequence a total of 12 gene segments for 24 representative accessions. At a first glance, the total PIC value of the 54 Set2 SNPs, for being in 12 tightly linked groups, must be lower than that of the same number of independent SNPs, but in fact the high SNP density also increased the haplotype diversity. The resulted PI value suggested that for the purpose of cultivar identification the 12 gene segments were already powerful enough. It must be pointed out that we did not intentionally select the target segments, yet they showed a surprisingly high discrimination power. Therefore, it should be possible to select even fewer high PIC segments to identify pummelo cultivars. Apparently, more DNA segment sequences are needed to identify pummelo cultivars compared to the DNA barcoding technology used in species identification that uses only 1 or a few DNA segment sequences [28], [29], [30] for the genetic differences are smaller between cultivars than between species.

For fruit trees like citrus, the genetic difference may be either very large between cultivars derived from inter-specific or inter-genic hybridizations or very small between cultivars originated from somatic mutations [66], [67]. It is easy to identity inter-specific or inter-genic hybrids but usually not easy to identify somatic mutants for it is almost impossible to locate by chance the specific point mutations when using a limited number of markers. As an example, the three mutants of the white flesh Guanximiyou, red flesh/white sponge Guanximiyou, red flesh/red sponge Sanhongyou and yellow flesh/white sponge Huangjinyou, were genotypically identical to white flesh Guanximiyou at all analyzed loci. Similarly, the mutant red Hassaku was identical to Hassaku. Beni Amanatsu and Kawano Natsudaidai were identical as expected for both were mutants of Natsudaidai. The grapefruits, ‘Red Marsh’, ‘Star Ruby’ and ‘Flame’ were also not surprisingly identified to have the same genotype since almost all grapefruit lineage can always be traced back to “Duncan”, the oldest grapefruit cultivar [68]. Though red flesh Shatianyou and early red flesh Shatianyou were identified to have the same genotype, they were not somatic mutations of any other SHATIANYOU member. Except somatic mutants, samples with identical genotypes should be regarded as synonyms. Examples are four SHATIANYOU cultivars, Gulaoqian, Lingnanshatianyou, Hejiangyou and Zhenlongyou that are known to be morphologically inseparable. Six groups of samples were identified as homonyms after verification with repeated genotyping,however, the possibility that they have been mixed during germplasm collection could not be excluded. Particularly, the three Huazhoujuhong accessions (ID 162, 163 and 176 in Table S1) were in fact different and should be treated as homonyms, and the results verified the notion that Huazhoujuhongs are a group of hybrids [69].

The origin and dispersion of the pummelo species remain controversial for no wild pummelos have been unequivocally recognized. South China, Southeast Asia and Northeast India were independently proposed to be the origin place since pummelos were highly polymorphic in these areas [1], [2], [3], [4], [70], [71]. Yunnan was suggested to be the origin center of citrus for being rich in not only native citrus species but also river systems that could facilitate the dispersion of citrus [72]. This may be especially true regarding the origin and dispersion of pummelo because pummelo fruit, for having peels with very thick sponge tissue to facilitate floating and enhance tolerance to mechanical injuries, could be carried away by rivers for long distances. It can be envisaged that pummelos were accidentally brought to the downstream by rivers originating from or flowing through Yunnan in ancient times, and then dispersed to nearby areas by fruit-eating animals to develop eventually isolated populations. These isolated populations served as genetic pools for domestication and should have been gradually mixed up. In this study, an obvious population structure was indeed revealed by Set1 SNPs. In addition, high haplotype diversity and high nucleotide diversity were observed on 12 gene segments of the 24 representative cultivars. Furthermore, a large number of the pummelo genotypes were inferred to be admixtures of different populations (Figure S2). It should also be mentioned that a lot of pummelos recorded in ancient Chinese literatures were distributed along the Yangtze River and the Pearl River that flow through Yunnan, and that pummelos were also found along riverbanks in Yunnan and Myanmar during our field investigations.

## Supporting Information

Figure S1 **Verification of the primer-template mismatch suspected in HRMA analysis (bottom panels in** **Figure 1** **) by sequencing.** A. Direct sequencing of one homozygote (Ho1) and the two heterozygotes (He1 and He2) on SNP chr4_18094735A/G (black arrows). The forward and reverse HRMA primers were indicated by blue and green arrows, respectively. A previously unknown SNP (chr4_18094754T/C, indicated by red arrows) was discovered in the reverse primer region and He2 was found to be heterozygous for this SNP. B. Diagrams showing how the extra SNP (chr4_18094754T/C)identified in panel A influenced the PCR amplification efficiency of He2 template. Three haplotypes (Ha1, Ha2 and Ha3) were reconstructed for chr4_18094735A/G and chr4_18094754T/C from analyzing melting curves and sequencing data. Ha3 was also identified in the sweet orange reference genome. The SNP induced mismatch on Ha3 (marked by a red cross) reduced the primer-template annealing temperature and thus reduced the PCR amplification efficiency for the He2.
(TIFF)

Figure S2 **Assignment of 156 pummelo accessions (CUL+UNKNOWN+MYANMAR) to four populations by STRUCTURE version 2.3.4.** P1, P2, P3, and P4 were represented by blue, violet, green and red, respectively. Accession IDs were the same as those in Table S1.
(TIFF)

Figure S3 **Neighbour-joining trees based on inferred pummelo haplotypes on 12 gene segments.** Nanju was used as a representative of mandarin. Inferred pummelo haplotypes and mandarin haplotypes were marked in green and orange, respectively. On the top of each tree was the gene ID designated in sweet orange reference genome [38]. Note: 1) Trees of Cs4g15590, Cs9g14320 and Cs7g10980 showed that pummelos contained mandarin haplotypes. 2) On tree of Cs5g15460, Iwaikan2 and Tangyouzi2 were identified as non-mandarin and non-pummelo haplotypes.
(PDF)

Table S1 178 genotypes of the 260 accessions on the 25 Set1 SNPs. Note: Red and blue background indicate the UNKNOWN group and the CUL group, respectively.
(XLSX)

Table S2 PI, PIpar-off and PIsib for all 178 genotypes. Note: PI for a given genotype is the probability that a randomly sampled individual is identical to the given genotype, as is the same case for PIsibs and PIpar-off [41].
(XLSX)
