# Correction: Identification of Pummelo Cultivars by Using a Panel of 25 Selected SNPs and 12 DNA Segments

**DOI:** 10.1371/journal.pone.0103375

**Published:** 2014-07-17

**Authors:** The PLOS ONE Staff

There is an error in the author affiliations for this article. Please refer to the correct author affiliations below:

Bo Wu ^1,2,3^, Guang-yan Zhong ^2,3*^, Jian-qiang Yue ^4^, Run-ting Yang ^1^, Chong Li ^1^, Yue-jia Li ^2,3^, Yun Zhong ^2,3^, Xuan Wang ^1^, Bo Jiang ^2,3^, Ji-wu Zeng ^2,3^, Li Zhang ^2,3^, Shu-tang Yan ^1^, Xue-jun Bei ^1^, Dong-guo Zhou ^4^

1 College of Horticulture and Landscape Architecture, Southwest University, Chongqing, China

2 Key Laboratory of South Subtropical Fruit Biology and Genetic Resource Utilization, Ministry of Agriculture, Guangzhou, China

3 Institution of Fruit Tree Research, Guangdong Academy of Agricultural Sciences, Guangzhou, China

4 Institute of Tropical and Subtropical Cash Crops, Yunnan Academy of Agricultural Science, Dehong, Yunnan, China
