# Supplementary figures and images for: Identification of Pummelo Cultivars by Using a Panel of 25 Selected SNPs and 12 DNA Segments
Source: PLoS One. 2014 Apr 14;9(4):e94506. doi: 10.1371/journal.pone.0094506 (PMC3986212; doi:10.1371/journal.pone.0094506)

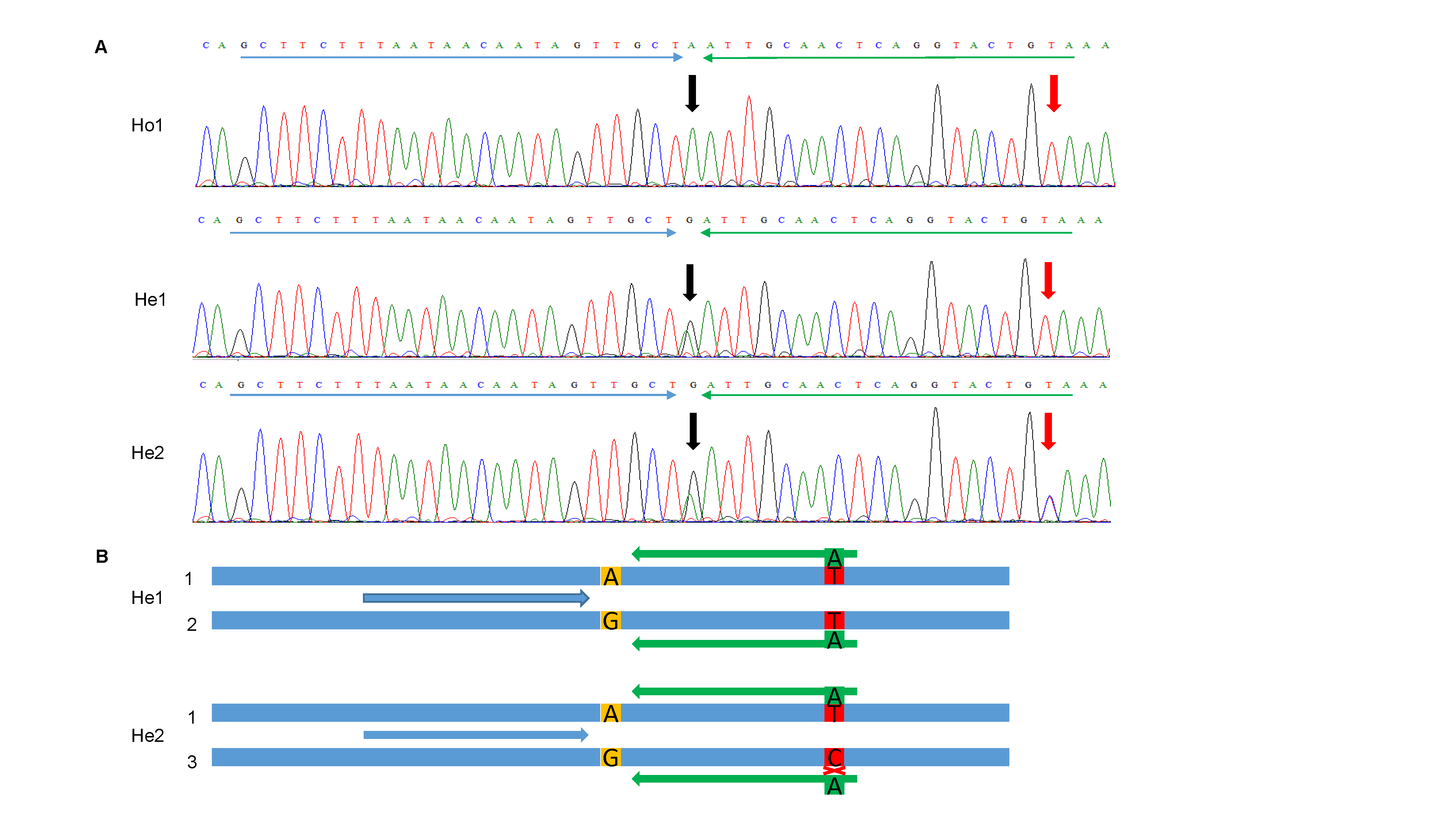

Supplement: Figure S1 — Verification of the primer-template mismatch suspected in HRMA analysis (bottom panels in Figure 1 ) by sequencing. A. Direct sequencing of one homozygote (Ho1) and the two heterozygotes (He1 and He2) on SNP chr4_18094735A/G (black arrows). The forward and reverse HRMA primers were indicated by blue and green arrows, respectively. A previously unknown SNP (chr4_18094754T/C, indicated by red arrows) was discovered in the reverse primer region and He2 was found to be heterozygous for this SNP. B. Diagrams showing how the extra SNP (chr4_18094754T/C)identified in panel A influenced the PCR amplification efficiency of He2 template. Three haplotypes (Ha1, Ha2 and Ha3) were reconstructed for chr4_18094735A/G and chr4_18094754T/C from analyzing melting curves and sequencing data. Ha3 was also identified in the sweet orange reference genome. The SNP induced mismatch on Ha3 (marked by a red cross) reduced the primer-template annealing temperature and thus reduced the PCR amplification efficiency for the He2. (TIFF) [file pone.0094506.s001.tif]

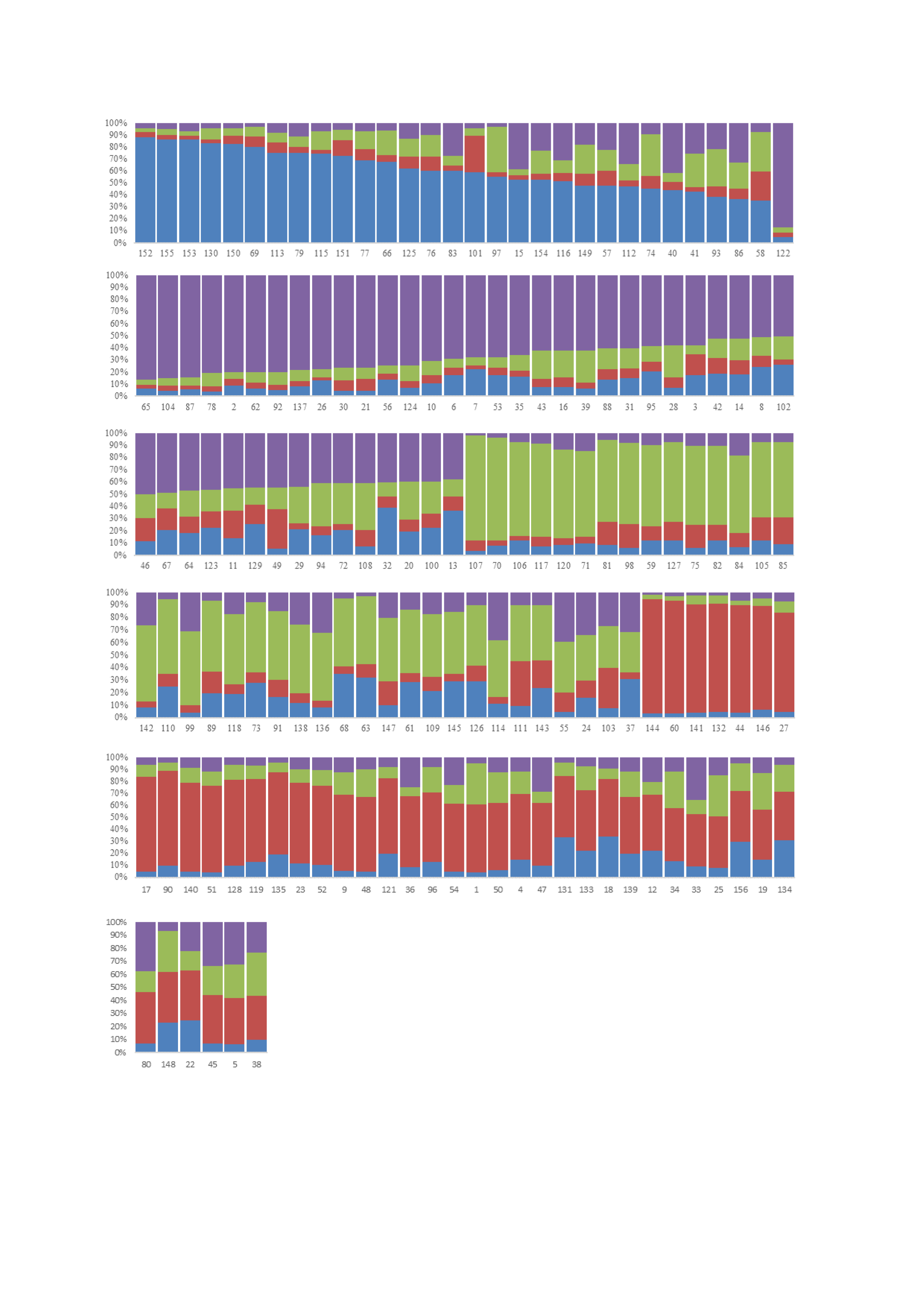

Supplement: Figure S2 — Assignment of 156 pummelo accessions (CUL+UNKNOWN+MYANMAR) to four populations by STRUCTURE version 2.3.4. P1, P2, P3, and P4 were represented by blue, violet, green and red, respectively. Accession IDs were the same as those in Table S1. (TIFF) [file pone.0094506.s002.tif]

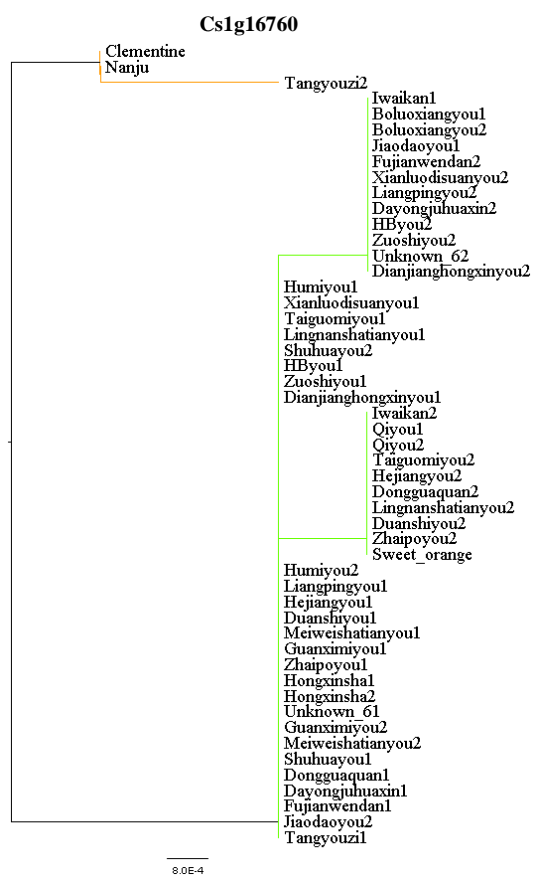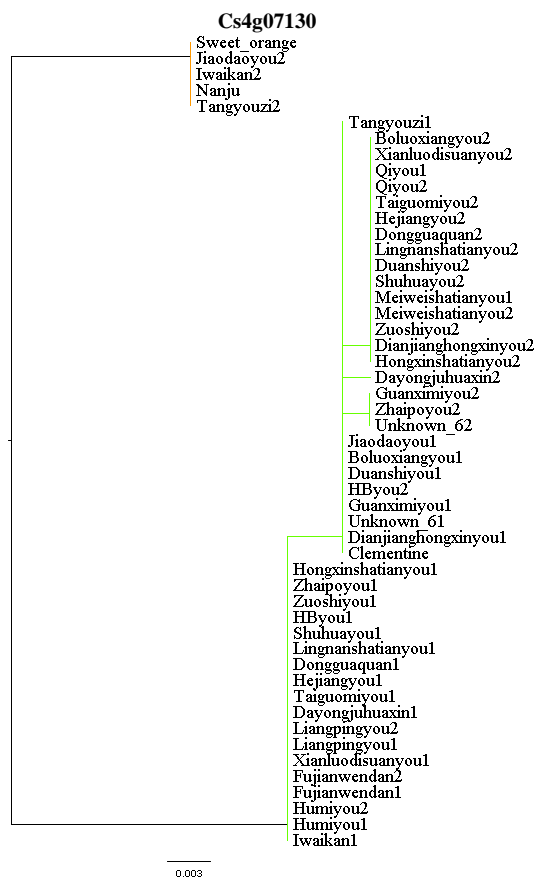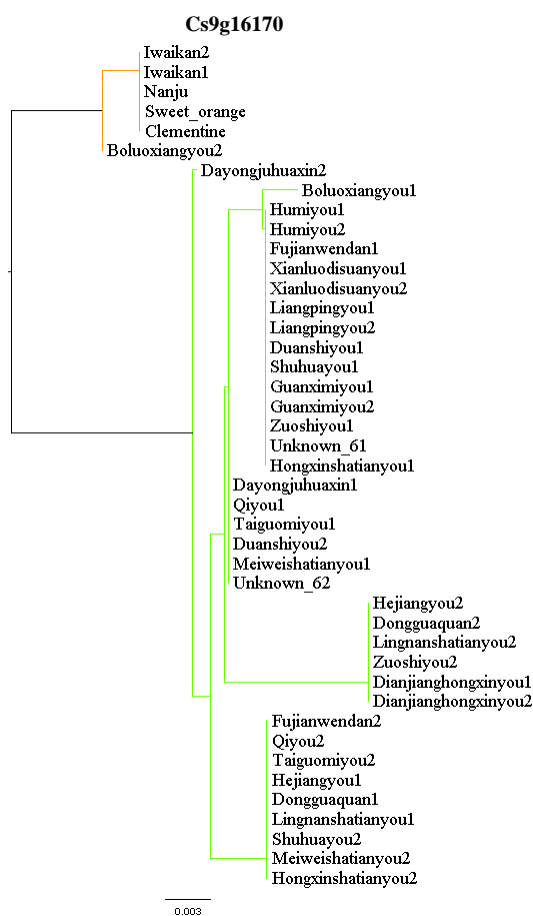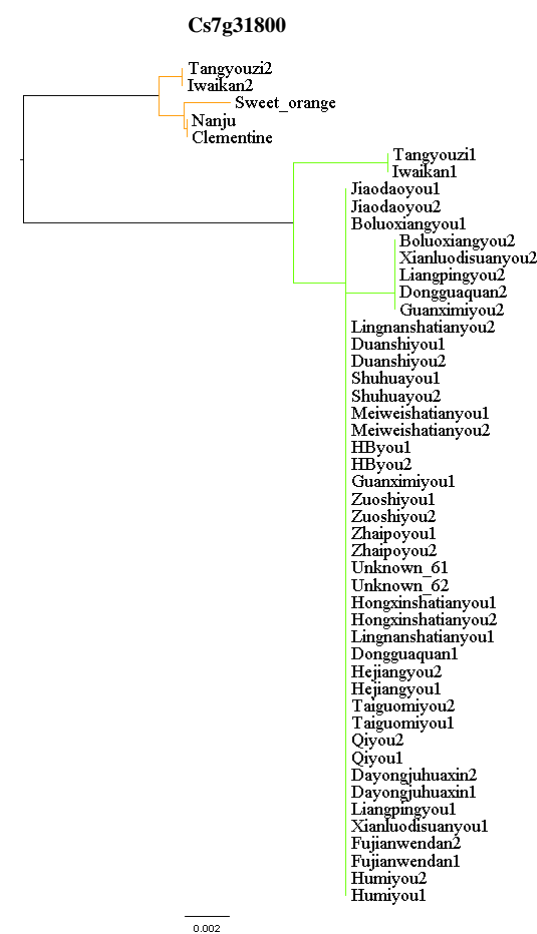

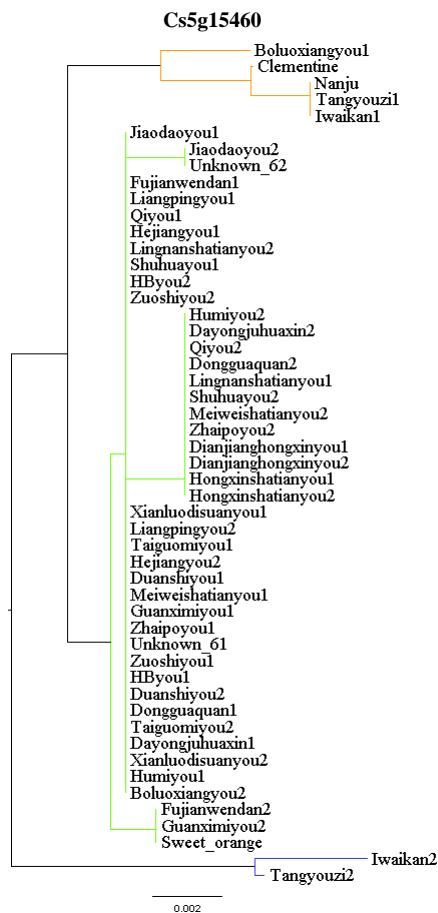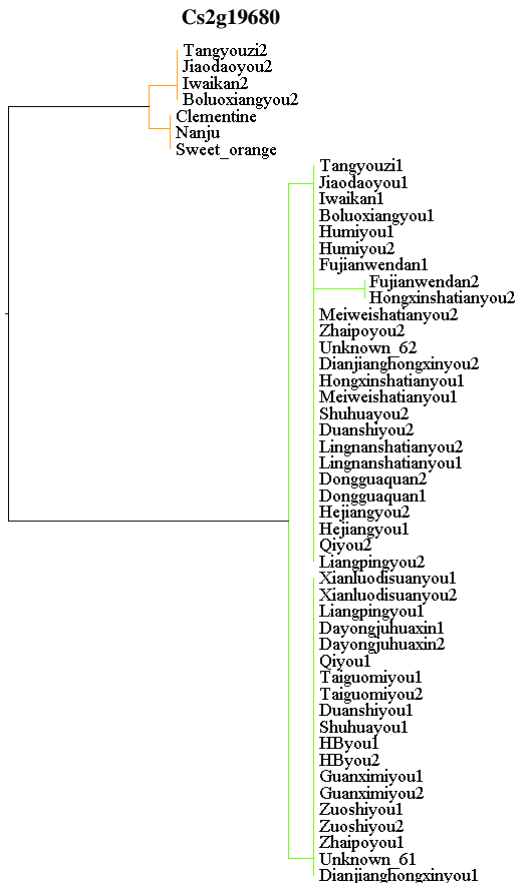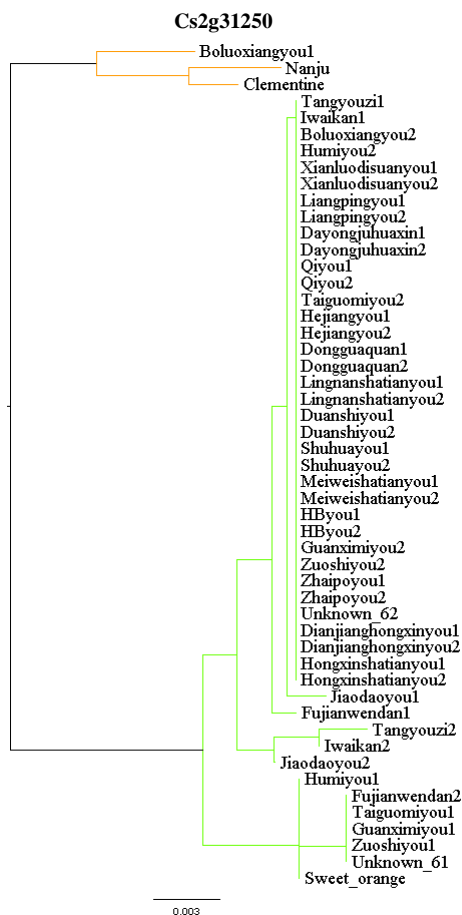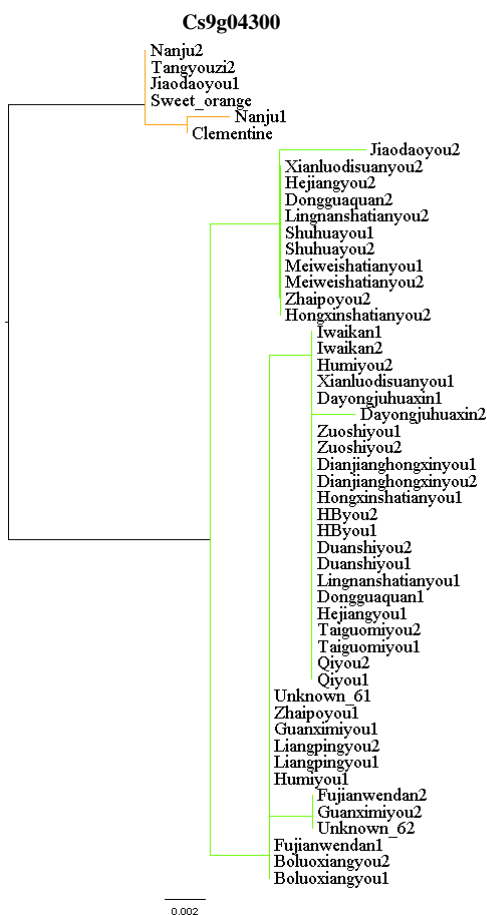

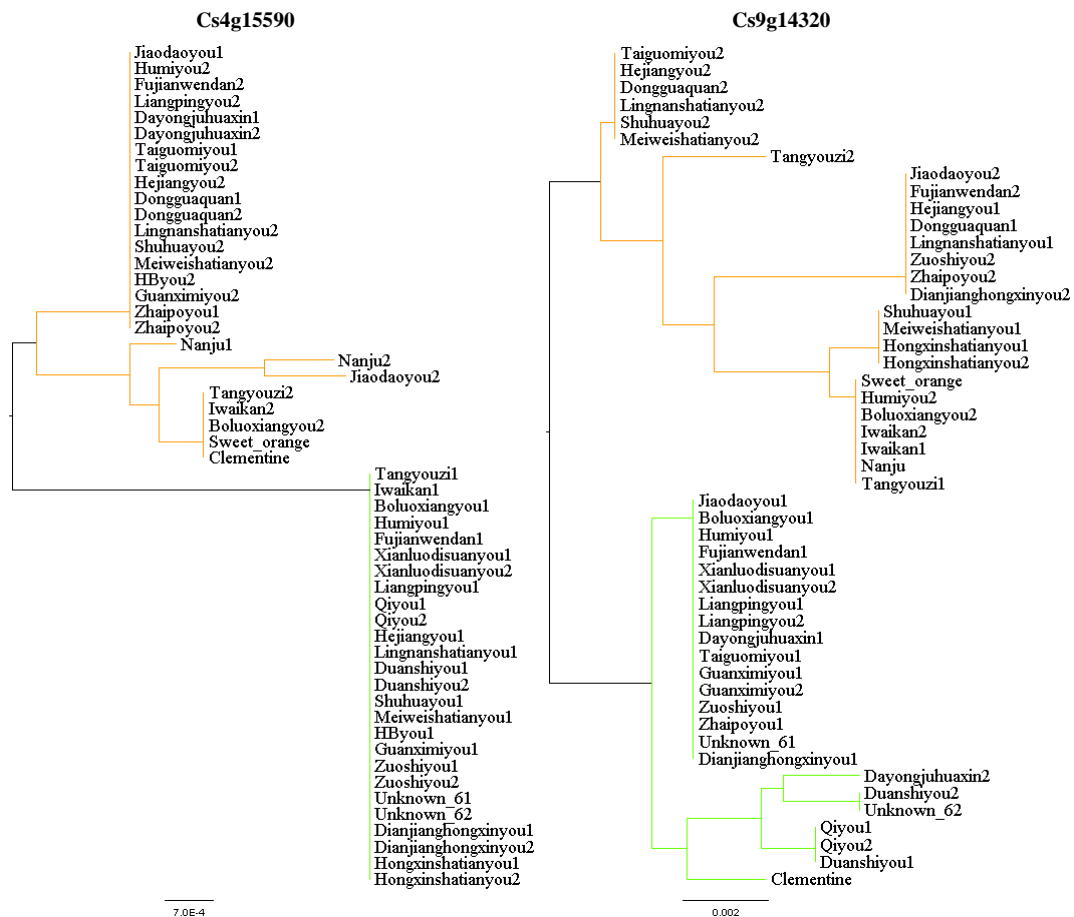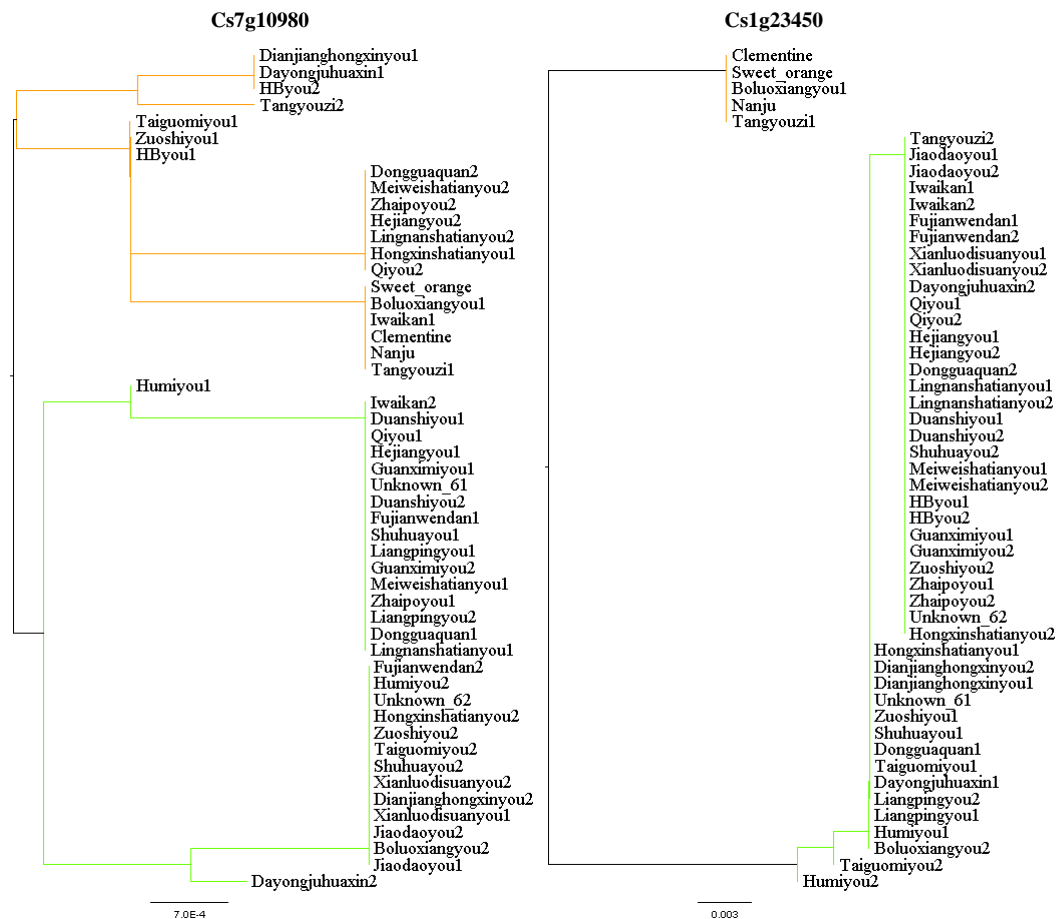

Supplement: Figure S3 — Neighbour-joining trees based on inferred pummelo haplotypes on 12 gene segments. Nanju was used as a representative of mandarin. Inferred pummelo haplotypes and mandarin haplotypes were marked in green and orange, respectively. On the top of each tree was the gene ID designated in sweet orange reference genome [38]. Note: 1) Trees of Cs4g15590, Cs9g14320 and Cs7g10980 showed that pummelos contained mandarin haplotypes. 2) On tree of Cs5g15460, Iwaikan2 and Tangyouzi2 were identified as non-mandarin and non-pummelo haplotypes. (PDF) [file pone.0094506.s003.pdf]
